# Effect of a Web-Based Behavior Change Program on Weight Loss and Cardiovascular Risk Factors in Overweight and Obese Adults at High Risk of Developing Cardiovascular Disease: Randomized Controlled Trial

**DOI:** 10.2196/jmir.3828

**Published:** 2015-07-16

**Authors:** Sinead Watson, Jayne V Woodside, Lisa J Ware, Steven J Hunter, Alanna McGrath, Christopher R Cardwell, Katherine M Appleton, Ian S Young, Michelle C McKinley

**Affiliations:** ^1^ Centre for Public Health School of Medicine, Dentistry and Biomedical Sciences Queen’s University Belfast Belfast United Kingdom; ^2^ Institute for Global Food Security School of Biological Sciences Queen’s University Belfast Belfast United Kingdom; ^3^ Hypertension in Africa Research Team (HART) Faculty of Health Sciences North-West University Potchefstroom South Africa; ^4^ Regional Centre for Endocrinology and Diabetes Royal Victoria Hospital Belfast United Kingdom; ^5^ Department of Psychology Faculty of Science and Technology Bournemouth University Poole United Kingdom

**Keywords:** Internet, randomized controlled trial, health behavior, weight loss, overweight, obesity

## Abstract

**Background:**

Web-based programs are a potential medium for supporting weight loss because of their accessibility and wide reach. Research is warranted to determine the shorter- and longer-term effects of these programs in relation to weight loss and other health outcomes.

**Objective:**

The aim was to evaluate the effects of a Web-based component of a weight loss service (Imperative Health) in an overweight/obese population at risk of cardiovascular disease (CVD) using a randomized controlled design and a true control group.

**Methods:**

A total of 65 overweight/obese adults at high risk of CVD were randomly allocated to 1 of 2 groups. Group 1 (n=32) was provided with the Web-based program, which supported positive dietary and physical activity changes and assisted in managing weight. Group 2 continued with their usual self-care (n=33). Assessments were conducted face-to-face. The primary outcome was between-group change in weight at 3 months. Secondary outcomes included between-group change in anthropometric measurements, blood pressure, lipid measurements, physical activity, and energy intake at 3, 6, and 12 months. Interviews were conducted to explore participants’ views of the Web-based program.

**Results:**

Retention rates for the intervention and control groups at 3 months were 78% (25/32) vs 97% (32/33), at 6 months were 66% (21/32) vs 94% (31/33), and at 12 months were 53% (17/32) vs 88% (29/33). Intention-to-treat analysis, using baseline observation carried forward imputation method, revealed that the intervention group lost more weight relative to the control group at 3 months (mean –3.41, 95% CI –4.70 to –2.13 kg vs mean –0.52, 95% CI –1.55 to 0.52 kg, *P*<.001), at 6 months (mean –3.47, 95% CI –4.95 to –1.98 kg vs mean –0.81, 95% CI –2.23 to 0.61 kg, *P*=.02), but not at 12 months (mean –2.38, 95% CI –3.48 to –0.97 kg vs mean –1.80, 95% CI –3.15 to –0.44 kg, *P*=.77). More intervention group participants lost ≥5% of their baseline body weight at 3 months (34%, 11/32 vs 3%, 1/33, *P*<.001) and 6 months (41%, 13/32 vs 18%, 6/33, *P*=.047), but not at 12 months (22%, 7/32 vs 21%, 7/33, *P*=.95) versus control group. The intervention group showed improvements in total cholesterol, triglycerides, and adopted more positive dietary and physical activity behaviors for up to 3 months verus control; however, these improvements were not sustained.

**Conclusions:**

Although the intervention group had high attrition levels, this study provides evidence that this Web-based program can be used to initiate clinically relevant weight loss and lower CVD risk up to 3-6 months based on the proportion of intervention group participants losing ≥5% of their body weight versus control group. It also highlights a need for augmenting Web-based programs with further interventions, such as in-person support to enhance engagement and maintain these changes.

**Trial Registration:**

ClinicalTrials.gov NCT01472276; http://clinicaltrials.gov/ct2/show/study/NCT01472276 (Archived by Webcite at http://www.webcitation.org/6Z9lfj8nD).

## Introduction

The prevalence of obesity has been increasing progressively throughout the world [1]. Identifying effective and cost-effective treatment and prevention strategies is a top priority for all health care systems. Over the past few decades, the Internet has increasingly been used to deliver behavioral modification programs owing to its easy accessibility and anonymity, potential for wide reach and penetration, and its ability to provide a source of continuous support to large segments of the population [2-4].

There is growing evidence suggesting that the Internet may be a viable medium for encouraging weight loss. However, several systematic reviews and meta-analyses, conducted in this area have found it difficult to draw definitive conclusions regarding its effectiveness owing to heterogeneity in study designs, methods employed, and the lack of “true control” groups used [5-9]. Most of the evidence to date comes from randomized controlled trials (RCTs) conducted in the United States. Many only included short-term follow-up and lacked true control groups (no support provided), making it challenging to accurately evaluate the true effectiveness of Web-based programs. Instead, minimal support groups are often employed to help boost recruitment and decrease attrition; although this approach may attenuate the relationship between groups and limits the ability of the findings to inform cost-effectiveness and health care models. Research is also limited regarding the effect of these Web-based programs on other health outcomes that coexist with weight loss, such as cardiovascular disease (CVD) risk factors. Therefore, the aim of this study was to evaluate the effects of an interactive Web-based component of a service called Imperative Health on weight loss (primary outcome) and CVD risk factors (secondary outcomes) in an overweight and obese population at high risk of CVD using a randomized controlled design and a true control group. It was hypothesized that weight loss would be greater in the Web-based program intervention group compared to the usual care control group.

## Methods

### Recruitment

Ethical approval was obtained from the Office for Research Ethics Committees Northern Ireland. The trial was registered (ClinicalTrials.gov identifier: NCT01472276) and is reported in accordance with the Consolidated Standards of Reporting Trials (CONSORT)-eHealth checklist (see Multimedia Appendix 1) [10]. Participants were recruited from April to December 2011 using posters in public places in the greater Belfast area and intranet advertisements via staff updates in the Belfast Health and Social Care Trust and Queen’s University Belfast (QUB). Patients from the Regional Centre for Diabetes and Endocrinology at the Royal Victoria Hospital Belfast were also sent a letter informing them about the study. Participants were eligible if they were older than 18 years, had a body mass index (BMI) between 27 and 40 kg/m^2^, were inactive or moderately inactive assessed by the General Practice Physical Activity Questionnaire (GPPAQ) [11] and had 1 or more CVD risk factors: high blood pressure ≥140/90 mmHg, total cholesterol ≥5.0 mmol/L, or type 2 diabetes mellitus. All participants were required to have access to the Internet, email, and a telephone and were asked not to participate in another behavioral change weight loss program throughout the study period. Participants were excluded if they had established CVD, type 1 diabetes mellitus, were pregnant, or consumed excessive amounts of alcohol. Computer literacy was not assessed. All participants at the screening appointment provided written informed consent.

### Study Design

After completion of the baseline assessments, conducted face-to-face at the Regional Centre for Diabetes and Endocrinology at the Royal Victoria Hospital Belfast, participants were randomly allocated to 1 of 2 parallel groups (1:1 allocation ratio) using a block randomization approach (block size=10) with computer-generated numbers. A researcher independent from the study prepared the randomization schedule. Opaque sealed envelopes were used to conceal the sequence until groups were allocated. Participants were recruited and enrolled by the researcher, who was unaware of the randomization schedule until after the baseline assessments when the sealed envelope containing the allocation outcome was opened by the participant. Group 1 (intervention group) was provided with the Web-based program, known as Imperative Health*,* excluding telephone and email support and group 2 (control group) were requested to continue with their usual self and medical care. All participants were followed up at 3, 6, and 12 months after randomization for assessment of primary and secondary outcomes. Based on a standard deviation of weight loss at 3 months of 3.0 kg observed in a number of Internet-based weight loss studies in the literature [12-15], it was estimated that a sample size of 60 (30 per group) would give the study 90% power at the 5% significance level to detect a difference of 2.6 kg between groups at the 3 month follow-up. Allowing for a 10% dropout rate at the first 3-month follow-up, we aimed to recruit 66 participants. With only one researcher on the ground, it was not possible to blind the researcher or participants to group allocation, but laboratory analysis was performed blind.

### Intervention (Imperative Health Web-Based Program)

#### Overview

Imperative Health is a service owned by AXA PPP Healthcare Limited that consists of a Web-based program and human (email and telephone) support that assists in lifestyle change, with a particular focus on improving diet and nutrition, increasing physical activity, and managing weight and other CVD risk factors. It combines objective monitoring of weight and physical activity with automated, tailored feedback and support by physiologists by telephone and email. Previous versions of this Web-based program have been evaluated by Hurling et al [16,17] and Ware et al [18]. This program has since been modified to be more relevant to individuals with independent risk factors for CVD, such as hypertension, dyslipidemia (high cholesterol and triglycerides), and type 2 diabetes mellitus. For this particular study, only the Web-based program component of the service was evaluated to determine its specific impact (ie, the human support [telephone and email] component of the service was removed for the purposes of this trial).

#### Initial Setup of Imperative Health (Web-Based Program)

At the end of the baseline appointment, the intervention group participants were provided with the Imperative Health package that contained the self-monitoring devices (Bluetooth-enabled weighing scales and an accelerometer activity band) and basic written instructions to set up an online account at home. To access the online program, participants were instructed to go to the Imperative Health website [19] and enter a unique code to create their own personal password-protected free account. Participants were advised to follow the online instructions to complete registration and to enable the setup of the monitoring devices. The intervention group was informed at the baseline appointment that if any problems regarding the technology occurred throughout the study period after the initial setup, they were to contact Imperative Health rather than the researcher. This study wanted to evaluate this Web-based program in a real-life setting to determine realistic levels of engagement and their relationship with weight loss; therefore, no instructions were provided by the researcher as to how often the participants should log in to use the website components and the self-monitoring devices. The Web-based program, however, does encourage daily engagement by allowing the upload of daily weight and physical activity data and by the entry of daily food diaries (described in detail subsequently).

#### Web-Based Behavior Change Program

Once the online account was set up, the participants were required to complete a series of online introductory health questionnaires that enabled Imperative Health to collect information on their height, weight, waist circumference, blood pressure and blood biomarkers (total cholesterol, high-density lipoprotein [HDL] cholesterol, fasting blood glucose, and triglycerides), as well as information on past and current health status, dietary intake, physical activity level, and stated goals. This self-reported information was not used by the researcher to evaluate the effects of this Web-based program; instead, it was used by the Imperative Health system to generate personalized daily targets (weight loss, physical activity, and dietary targets) for each participant to achieve over 12 weeks. Automated weekly feedback on their performance, assessed by the self-monitoring devices (weighing scales and accelerometer) and the food diary was provided, and also in the form of an overall review after 12 weeks. After 12 weeks, to encourage further progress, it was requested that the participants start a new program by completing the same introductory health questionnaires again and setting new goals. The Web-based program encompassed supportive components to help facilitate lifestyle change (see Table 1). These components of the Web-based program were developed based on well-recognized behavior change strategies, such as planning, self-monitoring, goal setting, and structured feedback, which were all used within the Diabetes Prevention Program [20] to promote weight loss.

**Table 1:** Imperative Health Web-based program components to support behavior change.

Behavior change strategy and Web-based program component		Description of component
**Goal setting**		
	Daily dietary targets, daily physical activity targets, weekly weight loss targets, clinical targets	Personalized daily dietary, physical activity (for screenshots see Figure 1), weight, and clinical (blood pressure, glucose, lipids) targets were created based on the health questionnaire responses. Targets were reviewed every 12 weeks.
**Planning**		
	Exercise weekly schedule	A weekly schedule for planning physical activity was provided. Icons (representing light, moderate, or vigorous activities) could be dragged to specific days. Start times and duration of the activity could be selected (for screenshots see Figure 1).
	Daily meal planners	Meal suggestions for breakfast, lunch, dinner, and snacks were provided to help meet personalized dietary targets set by the Web-based program.
**Self- monitoring**		
	Bluetooth weighing scales, Bluetooth accelerometer activity band	Monitoring devices included Bluetooth-enabled weighing scales and an activity band. Data from the weighing scales was transmitted to the activity band and subsequently sent to the user’s online profile page. The activity band provided daily feedback on minutes of moderate, high, and very high activity (for screenshots see Figure 2).
	Food diary (calorie uploads), clinical measurements (blood pressure, glucose, blood lipids uploads)	Daily calorie intake, blood pressure, glucose, and blood lipid measurements could be entered and uploaded onto colored charts (for screenshots see Figures 2 and 3) to demonstrate daily, weekly, and monthly results and if targets were achieved.
**Personalized feedback**		
	Coaching session, automated weekly feedback	Automated tailored feedback on progress was provided weekly.
**Push reminders**		
	Email/SMS texts	Text messages or emails were sent daily and weekly to help remind participants to log in and to weigh themselves.
**Social support**		
	Community forum	Online discussion forums were available for comments to be posted.
**Decisional balance theory**		
	Habit breaker component	Solutions for barriers perceived as preventing healthier behaviors (eg, eating breakfast) being adopted were provided.

**Figure 1:**
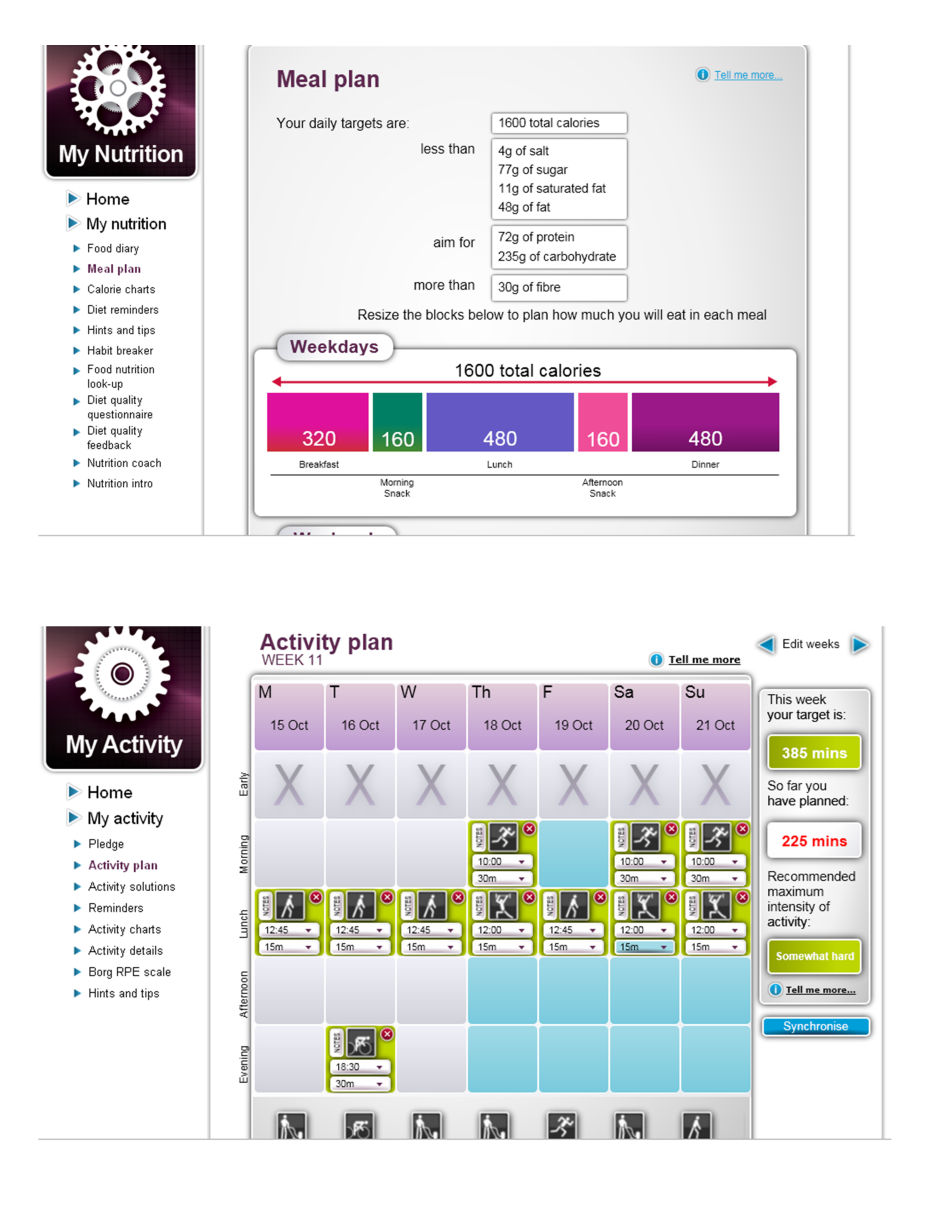
Imperative Health screenshots of meal and activity planners.

**Figure 2:**
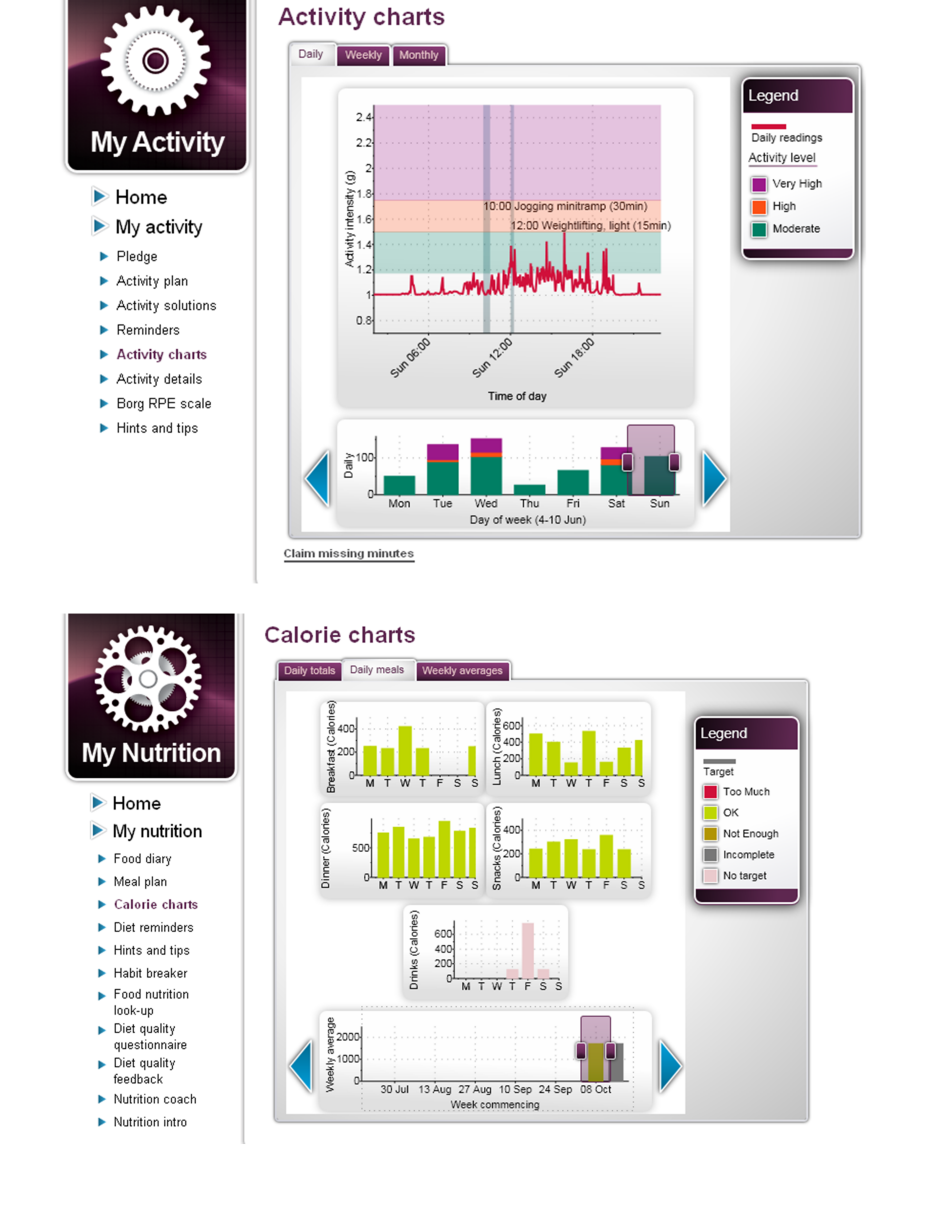
Imperative Health screenshots of activity and calorie feedback charts.

**Figure 3:**
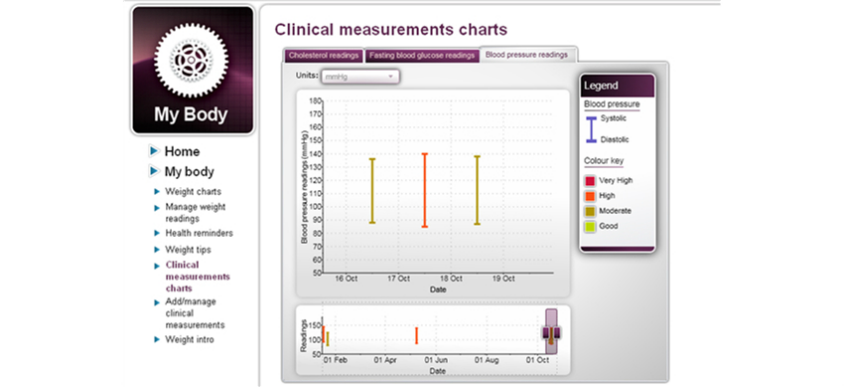
Imperative Health screenshot of clinical measurement feedback chart.

### Outcome Measures

#### Overview

Assessments were carried out face-to-face at the Regional Centre for Endocrinology and Diabetes at the Royal Victoria Hospital Belfast at baseline, 3 months, 6 months, and 12 months.

#### Primary Outcome

The primary outcome for this study was between-group change in body weight (kg) at 3 months. Weight was measured, without shoes and in light clothing, to the nearest 0.1 kg using calibrated Salter 994 digital weighing scales (Salter Housewares Ltd, Tonbridge, UK).

#### Secondary Outcomes

Secondary outcomes were between-group change in weight loss at 6 and 12 months, and between-group change in the following risk markers at each follow-up: BMI calculated as weight (kg) divided by height squared (m^2^); height was measured to the nearest 0.1 cm using a Leicester portable height measure (CMS Weighing Equipment Ltd, London, UK); waist circumference was measured to the nearest 0.5 cm using a tape measure at the middle point between the lower rib margin and iliac crest at normal expiration.

Blood pressure (mm Hg) was measured using an automated Omron M3 sphygmomanometer (Omron Healthcare, Hoofddorp, The Netherlands).

Fasting serum lipid profile included measurements of total cholesterol, HDL cholesterol, and triglycerides and were measured using standard assays on an automated ILab 600 Chemistry system (Instrumentation Laboratory, Cheshire, UK). Plasma high-sensitivity C-reactive protein (hs-CRP) was measured using an ultrasensitive assay (Quantex CRP Ultrasensitive; Instrumentation Laboratory, Cheshire, UK) on an automated machine (ILab 600 Chemistry System).

Dietary intake was assessed using a diet history interview [21], which was a retrospective dietary assessment method used to gather information regarding the habitual food intake of all participants over the previous 3 months. The diet history method has been shown to have good repeatability in previous studies and is also able to pick up dietary changes over time [22]. Quantities of food and food portion sizes (household measures) were converted into weights (grams) by using Crawley’s Food Portion Sizes (Food Standards Agency) [23]. The food type, preparation method if relevant, and weight of food were entered into a computerized food analysis database (WISP, Weighed Intake Software Program; Tinuviel Software, Warrington, UK). For the purpose of this study, total daily energy intake (kcal) was calculated.

Physical activity was assessed using the validated Recent Physical Activity Questionnaire (RPAQ) [24]. Participants were asked to provide descriptions of their habitual physical activity performed in 4 domains: home, work, travel, and recreation over the last 4 weeks. For the purpose of this study, time (min/day) spent participating in moderate and vigorous activities (> 3.5 Metabolic Equivalent Task, MET) was calculated.

A self-reported questionnaire was distributed at the baseline appointment to collect sociodemographic information including past and current occupation. Socioeconomic status was classified according to National Statistics Socio-economic Classification (NE-SEC) [25] into 3 occupational classes: the highest included higher managerial, administrative, and professional occupations; the second class was intermediate occupations; and the third class included routine and manual occupations.

### Website Usage

Data on frequency of log-ins, the total number of completed food diaries, and the number of weight and physical activity uploads from the monitoring devices were provided by Imperative Health and were used to determine level of engagement.

### Qualitative: Interviews

To gain in-depth feedback on the intervention group’s experiences of using the Web-based program, these participants were asked if they would be willing to take part in an interview conducted by the researcher toward the end of the study. This was an optional part of the study; therefore, a convenience sampling technique was utilized. The interviews were conducted between July and August 2012, in the Centre for Public Health, QUB, within an informal setting and lasted approximately 25 to 30 minutes. Semistructured open-ended questions were used throughout to ensure that a consistent approach was utilized. The researcher used a style of probing to extract more information or clarify meaning.

All the interviews were audio-recorded and transcribed verbatim. NVivo 8 was used to assist in the management and analysis of the transcripts. To analyze the transcripts, a template approach, outlined by Crabtree and Miller [26], was utilized. This process involved the naming, defining, and describing of the codes based on research questions. Three broad categories formed the code template: views on their experiences of using Imperative Health (Web-based program), views on Imperative Health*’s* website components that support behavior change, and suggested improvements that Imperative Health should implement. The template of codes was then applied to all transcripts. Given that the data were qualitative, frequencies were used in the broadest sense (eg, majority, some, and few). Quotations were used to demonstrate typical views within each code category.

### Statistical Analysis

All analyses were performed using SPSS for Windows version 21.0 (SPSS Inc, Chicago, IL, USA). Results are expressed as mean and standard deviation for normally distributed variables and median and interquartile range for variables that did not satisfy normality criteria. Categorical data are expressed as frequencies and percentages. To compare baseline characteristics between the control and intervention groups, for continuous variables, the appropriate parametric (independent samples *t* test) and nonparametric tests (Mann-Whitney *U* test) were utilized. For categorical variables, the chi-square test was used. Between-group differences in the primary outcome (weight change at 3 months) and secondary outcomes from baseline to 3 months, 6 months, and 12 months were investigated using the analysis of covariance (ANCOVA) adjusted for baseline measurements [27]. Analyses were carried out by an intention-to-treat (ITT) approach using a single imputation method (baseline observation carried forward, BOCF) to deal with missing data and losses to follow-up [28]. A complete-case analysis on weight change was also conducted using information on all individuals with available data at each time point. A sensitivity analysis was conducted using MANOVA, but this reached similar findings (results not shown). Triglycerides, CRP, and physical activity distributions were skewed; therefore, they were log transformed. Adjusted differences in log-transformed means between groups from ANCOVA were converted to, and reported as, ratios of geometric means and 95% confidence intervals. Within-group changes (intervention or control) in weight loss were analyzed using paired-sample *t* tests. Because the Web-based program usage data was not normally distributed, Spearman correlations were performed to investigate the relationship between weight change and Web-based program usage at each time point (intervention group only).

## Results

### Participant Flow

**Figure 4:**
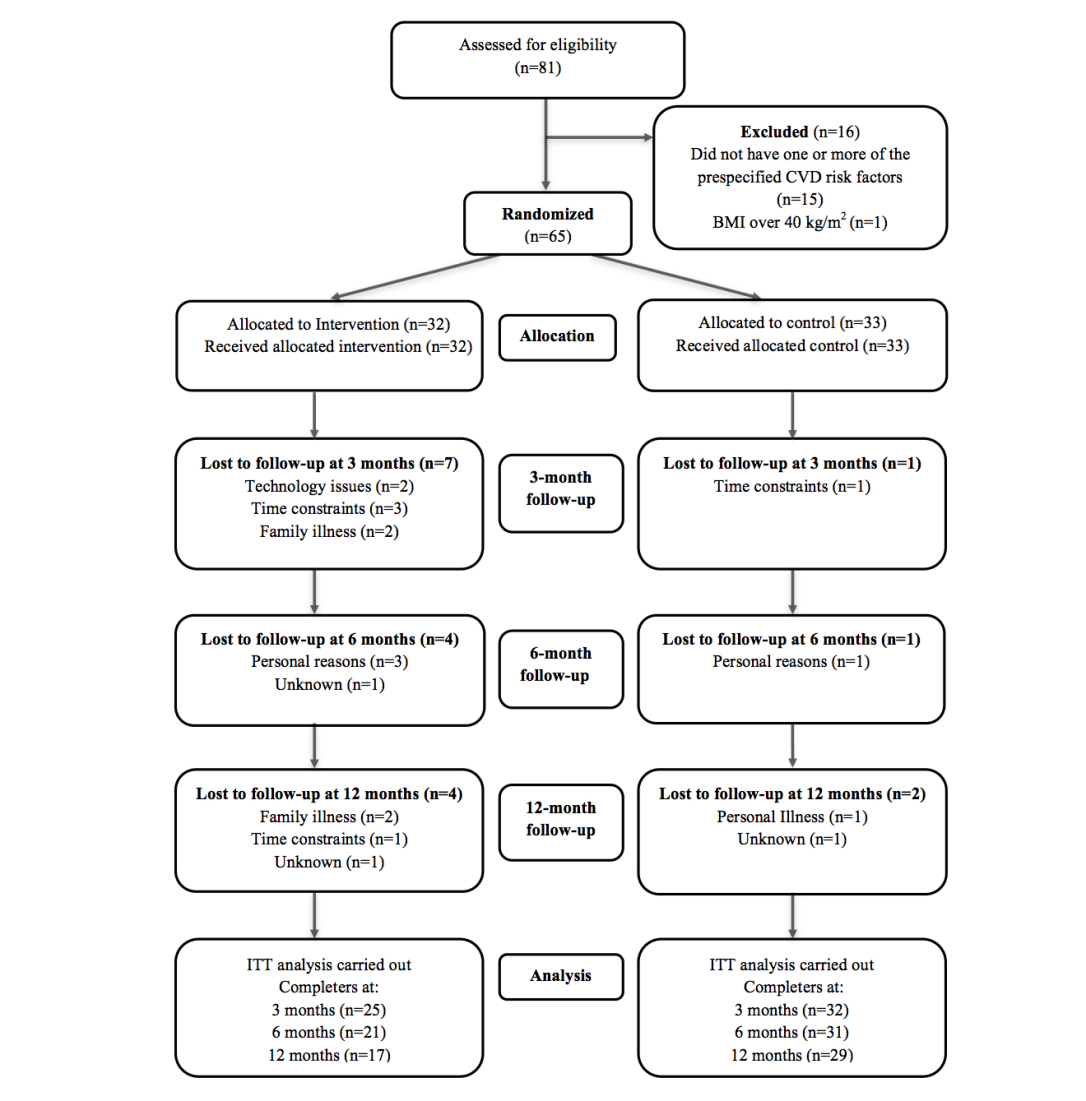
CONSORT diagram showing the flow of participants through the trial and analyzed for weight loss at 3 months, 6 months, and 12 months.

A total of 81 individuals were screened for eligibility; 16 were ineligible and the other 65 participants (29 males, 36 females) were randomized to the control (n=33) or intervention (n=32) groups (see Figure 4). Retention rates significantly differed between the control and intervention groups at 3 months (32/33, 97% vs 25/32, 78%, *P*=.03), at 6 months (31/33, 94% vs 21/32, 66%, *P*=.004), and at 12 months (29/33, 88% vs 17/32, 53%, *P*=.002), respectively. Baseline characteristics, including socioeconomic status, did not differ significantly between those who dropped out of the study and those who completed the study in either group at any time point.

### Baseline Characteristics

Mean age was 52.1 (SD 7.4) years and mean BMI at baseline was 32.7 (SD 2.9) kg/m^2^. According to World Health Organization criteria [29], 20% (13/65) of the sample were overweight, 58% (38/65) were obese category I, and 22% (14/65) were obese category II. Socioeconomic status (SES) was determined by occupational class (NS-SEC): Class 1 included higher managerial, administrative, and professional occupations, which 51% (33/65) of the sample lay within; Class 2 included intermediate occupations of which 38% (25/65) of the sample lay within; and Class 3 included routine and manual occupations, and applied to 11% (7/65) of the sample. There were no significant differences in any of the baseline characteristics between the control and intervention group (see Table 2).

**Table 2:** Baseline characteristics of intervention and control participants according to control and intervention group.

Characteristic^a^			Intervention (n=32)	Control (n=33)	*P* ^b^
**Demographics**					
	Gender, n (%)				.39
		Male	16 (50)	13 (39)	
		Female	16 (50)	20 (61)	
	Age (years), mean (SD)		51.4 (7.59)	52.9 (7.27)	.43
**Physical measurements, mean (SD)**					
	Weight (kg)		95.2 (16.7)	91.9 (13.4)	.39
	Height (cm)		169.4 (9.44)	168.1 (9.35)	.57
	BMI (kg/m^2^)		32.9 (3.07)	32.4 (2.74)	.50
	Waist circumference (cm)		103.5 (11.2)	102.5 (9.47)	.69
	**Blood pressure (mm Hg), mean (SD)**				
		Systolic	129.8 (17.8)	129.1 (18.3)	.88
		Diastolic	85.5 (9.54)	86.0 (11.4)	.85
	**Blood biomarkers**				
		Total cholesterol (mmol/L), mean (SD)	4.87 (1.44)	5.16 (1.02)	.35
		HDL (mmol/L), mean (SD)	1.33 (0.39)	1.36 (0.31)	.77
		Triglycerides (mmol/L), median (IQR)	1.49 (1.18-1.86)	1.48 (1.01-2.02)	.75
		CRP (mg/L), median (IQR)	1.73 (0.67-2.90)	2.11 (1.11-4.35)	.22
	**Energy intake and physical activity**				
		Energy (kcal), mean (SD)	1949.6 (545.1)	1893.6 (477.2)	.66
		Physical activity (min/day), median (IQR)	15.5 (6.4-45.3)	17.4 (7.5-46.9)	.72

^a^ BMI: body mass index; HDL: high-density lipoprotein cholesterol; CRP: C-reactive protein.

^b^ Between-group differences analyzed using independent samples *t* test for normal data and Mann-Whitney U test for skewed data. Differences between categories analyzed using chi-square test.

### Change in Body Weight (Primary Outcome)

As shown in Table 3, both approaches (ITT and complete case) demonstrated significant mean weight loss difference between groups at 3 months; however, the magnitude of weight lost was slightly higher using the complete-case analysis approach. ITT analysis revealed that the intervention group participants had a mean weight loss of –3.41 kg at 3 months; the control group lost –0.52 kg. Overall, this accounted for a significant mean weight difference between groups of –2.70 kg after adjusting for baseline weight (*P*<.001).

**Table 3:** Weight (kg) outcome differences between and within study groups from baseline to 3, 6, and 12 months (intention-to-treat [ITT] and complete-case analysis).

Analysis and month		Change from baseline, mean (95% CI)^a^		Difference between groups^b^	
		Intervention	Control	Mean (95% CI)	*P*
**ITT** ^c^					
	3	–3.41 (–4.70, –2.13)^***^	–0.52 (–1.55, 0.52)	–2.70 (–4.27, –1.13)	.001
	6	–3.47 (–4.95, –1.98)^***^	–0.81 (–2.23, 0.61)	–2.49 (–4.50, –0.48)	.02
	12	–2.38 (–3.48, –0.97)^**^	–1.80 (–3.15, –0.44)^*^	–0.27 (–2.16, 1.61)	.77
**Complete case** ^d^					
	3	–4.37 (–5.80, –2.94)^***^	–0.53 (–1.60, 0.54)	–3.66 (–5.28, –2.05)	<.001
	6	–5.28 (–7.12, –3.44)^***^	–0.86 (–2.38, 0.65)	–4.16 (–6.46, –1.86)	.001
	12	–4.48 (–7.34, –2.37)^**^	–2.16 (–4.58, –0.62)^*^	–1.89 (–4.42, 0.64)	.14

^a^ Within-group weight changes were analyzed using paired-sample *t* tests and only significant results are presented. ^*^ *P*<.05, ^**^ *P*<.01, ^***^ *P*<.001.

^b^ Difference between groups analyzed using ANCOVA and adjusted for baseline weight.

^c^ ITT analysis: control group (n=33) and intervention group (n=32) at 3, 6, and 12 months.

^d^ For the complete-case analysis: control group (n=32) at 3 months, (n=30) at 6 months, and (n=29) at 12 months. Intervention group (n=25) at 3 months, (n=21) at 6 months, and (n=17) at 12 months.

### Change in Body Weight at 6 and 12 Months (Secondary Outcome)

The ITT analysis (see Table 3) demonstrated that the intervention group lost significantly more weight compared to the control group from baseline to 6 months (mean –3.47, 95% CI –4.95 to –1.98 kg vs mean –0.81, 95% CI –2.23 to 0.61 kg; *P*=.02, respectively), but not from baseline to 12 months (mean –2.38, 95% CI –3.48 to –0.97 kg vs mean *–*1.80, 95% CI –3.15 to –0.44 kg; *P*=.77). There were significant changes in weight between baseline and each time point within the intervention group (3 months: *P*<.001; 6 months: *P*<.001; 12 months: *P*=.002). However, between 6 months and 12 months the intervention group gained 1.08 kg, reducing the overall mean weight loss at 12 months in this group. There was a significant weight loss from baseline to 12 months within the control group (mean –1.80, 95% CI –3.15 to –0.44 kg, *P*=.01), but not for the 3 month (*P*=.32) and 6 month (*P*=.25) time points.

### Percentage Weight Loss

Weight loss as a percentage of baseline weight was calculated using the ITT data. The mean percentage weight loss in the intervention and the control group was from baseline to 3 months mean –3.62% (95% CI –4.95 to –2.29) vs mean –0.34% (95% CI –1.34 to 0.65), respectively (*P*<.001); from baseline to 6 months mean –3.73% (95% CI –5.30 to –2.16) vs mean –0.63% (95% CI –2.06 to 0.80), respectively (*P*=.004); and from baseline to 12 months mean –2.42% (95% CI –3.93 to –0.91) vs –1.94% (95% CI –3.26 to –0.39), respectively (*P*=.56). Significantly more participants in the intervention group compared to the control group lost 5% or more of their baseline body weight at 3 months (11/32, 34% vs 1/33, 3%, *P*<.001) and at 6 months (13/32, 41% vs 6/33, 18%, *P*=.047), but not at 12 months (7/32, 22% vs 7/33, 21%, *P*=.95).

### Change in Other Secondary Outcomes

Table 4 shows the intervention group significantly reduced their BMI and waist circumference measurements relative to the control group from baseline to 3 months (*P*<.001 and *P*=.006, respectively) and to 6 months (*P*=.003 and *P*=.02, respectively), but not at 12 months. There were no between-group differences in blood pressure observed during the study. For lipid measurements, larger reductions were observed in total cholesterol and triglyceride concentrations in the intervention group compared to the control group, but only during the first 3 months (*P*=.003 and *P*=.003, respectively). Similar patterns were identified for health behaviors: the intervention group significantly decreased their energy intake and increased their time spent exercising at an intensity greater than 3.5 METs relative to the control group from baseline to 3 months (*P*=.005 and *P*=.03). These behaviors were not sustained over the longer term at 6 and 12 months.

**Table 4:** Clinical outcome differences between study groups from baseline to 3, 6, and 12 months (intention to treat).

Clinical outcome and month^a^			Change from baseline, mean (95% CI)		Between-group difference	
			Intervention (n=32)	Control (n=33)	Adjusted mean (95% CI)^b^	*P*
**BMI (kg/m^2^)**						
	3		–1.16 (–1.60, –0.73)	–0.14 (–0.47, 0.19)	–0.99 (–1.53, –0.46)	<.001
	6		–1.20 (–1.70, –0.70)	–0.18 (–0.64, 0.27)	–1.02 (–1.69, –0.35)	.003
	12		–0.78 (–1.26, –0.31)	–0.65 (–1.12, 0.19)	–0.10 (–0.75, 0.55)	.76
**Waist circumference (cm)**						
	3		–2.73 (–3.98, –1.49)	–0.67 (–1.44, 0.11)	–2.04 (–3.47, –0.61)	.006
	6		–3.05 (–4.68, –1.41)	–0.83 (–1.95, 0.28)	–2.18 (–4.11, –0.24)	.02
	12		–2.31 (–3.84, –0.79)	–1.80 (–3.02, –0.58)	–0.42 (–2.29, 1.45)	.66
**Systolic blood pressure (mm Hg)**						
	3		–2.69 (–6.48, 1.10)	–1.64 (–6.02, 2.75)	–0.81 (–5.61, 3.99)	.74
	6		–1.31 (–4.83, 2.20)	0.88 (–3.79, 5.55)	–1.92 (–6.48, 2.65)	.40
	12		–1.22 (–4.33, 1.90)	–2.12 (–2.25, 6.49)	–3.13 (–7.69, 1.43)	.18
**Diastolic blood pressure (mm Hg)**						
	3		–3.03 (–5.14, –0.92)	–2.36 (–5.02, 0.29)	–0.83 (–3.76, 2.10)	.58
	6		–2.63 (–5.05, –0.20)	–1.73 (–5.26, 1.81)	–1.14 (–4.55, 2.27)	.51
	12		–1.78 (–3.52, –0.05)	–1.55 (–4.57, 1.48)	–0.38 (–3.52, 2.76)	.81
**Total cholesterol (mmol/L)**						
	3		–0.49 (–0.70, –0.28)	–0.06 (–0.31, 0.19)	–0.48 (–0.79, –0.18)	.003
	6		–0.30 (–0.53, –0.08)	–0.24 (–0.46, –0.02)	–0.07 (–0.38, 0.24)	.64
	12		–0.19 (–0.38, –0.01)	–0.13 (–0.36, 0.10)	–0.09 (–0.38, 0.20)	.56
**HDL (mmol/L)**						
	3		–0.02 (–0.08, 0.04)	0.00 (–0.07, 0.07)	–0.03 (–0.11, 0.06)	.51
	6		–0.01 (–0.07, 0.06)	–0.03 (–0.10, 0.04)	0.02 (–0.07, 0.12)	.62
	12		–0.02 (–0.07, 0.02)	0.02 (–0.06, 0.10)	–0.04 (–0.13, 0.04)	.32
**Triglycerides (mmol/L)** ^c^						
	3		0.89 (0.82, 0.96)	1.03 (0.96, 1.10)	0.87 (0.80, 0.95)	.003
	6		0.96 (0.89, 1.04)	0.98 (0.91, 1.04)	0.99 (0.90, 1.09)	.79
	12		0.97 (0.91, 1.02)	0.97 (0.90, 1.05)	1.00 (0.92, 1.09)	.93
**CRP (mg/L)** ^c^						
	3		0.93 (0.84, 1.03)	1.01 (0.87, 1.18)	0.88 (0.74, 0.96)	.13
	6		0.89 (0.83, 0.95)	1.03 (0.86, 1.24)	0.83 (0.69, 1.01)	.06
	12		0.99 (0.80, 1.23)	0.99 (0.81, 1.21)	0.93 (0.71, 1.21)	.58
**Energy intake (Kcal)**						
	3		–487.6 (–640.7, –334.5)	–241.4 (–375.8, –106.9)	–216.3 (–364.0, –68.7)	.005
	6		–314.8 (–466.2, –163.5)	–243.2 (–393.6, –92.8)	–47.8 (–228.5, 133.0)	.60
	12		–221.6 (–363.5, –79.8)	–204.2 (–384.5, 23.9)	7.20 (–191.0, 205.4)	.94
**Physical activity (min/day)** ^**c,d**^						
	3		2.85 (1.64, 4.94)	1.43 (0.92, 2.21)	1.98 (1.09, 3.60)	.03
	6		1.43 (1.00, 2.06)	1.00 (0.61, 1.64)	1.43 (0.84, 2.44)	.19
	12		1.52 (0.95, 2.43)	1.18 (0.74, 1.89)	1.28 (0.72, 2.27)	.40

^a^ BMI: body mass index; HDL: high-density lipoprotein cholesterol; CRP: C-reactive protein.

^b^ Difference between groups analyzed using ANCOVA and adjusted for baseline measurements.

^c^ Data presented as ratio of geometric mean and 95% confidence for log-transformed variables.

^d^ Physical activity calculated as time in minutes spent exercising >3.5 METs daily.

### Website Usage and Weight Change (Intervention Group Only)

Website utilization data are presented in Table 5. Participants in the intervention group tended to log in, upload their weight measurement, and make food diary entries more frequently during the first 3 months of the intervention; website usage declined thereafter.

**Table 5:** Website utilization patterns (intervention group only) from baseline to 3, 6, and 12 months.

Website components	Baseline to 3 months (13 weeks), median (IQR)^a^	3 to 6 months (13 weeks), median (IQR)^a^	6 to 12 months (26 weeks), median (IQR)^a^
Number of log-ins	69.0 (25.5-122.0)	12.0 (2.0-47.5)	27.0 (2.0-96.8)
Food diary entries	18.0 (0.0-77.0)	0.0 (0.0-38.5)	0.0 (0.0-156.5)
Weight uploads	15.0 (9.0-46.0)	11.0 (1.0-30.0)	4.0 (0.0-29.8)
Physical activity uploads	13.0 (10.0-13.0)	12.0 (3.0-13.0)	15.0 (0.0-24.5)

^a^ Data presented as median (IQR) due to data being skewed. Sample size at 3 months (n=25), at 6 months (n=21), and at 12 months (n=17).

Correlation analyses (see Table 6) demonstrated that weight change from baseline to 3-month follow-up was significantly positively related to the number of log-ins (*P*=.04) and the number of weight uploads (*P*=.007) at 3 months. A positive relationship was observed between weight change from baseline to 6 months and the amount of physical activity uploads over the same time period (*P*=.048). The number of daily food diaries entered was not related to weight change throughout the course of the study.

**Table 6:** Spearman’s correlations (ρ) between weight change and website components usage from baseline (intervention group only).

Website component usage	Weight change from baseline					
	3 months		6 months		12 months	
	ρ	*P*	ρ	*P*	ρ	*P*
Number of log-ins	.42	.04	.28	.21	.21	.42
Food diary entries	.01	.96	.00	.99	–.20	.53
Physical activity uploads	.33	.14	.47	.048	.12	.67
Weight uploads	.53	.007	.20	.39	.10	.70

### Interview Feedback (Intervention Group Only)

#### Overview

A total of 7 participants (4 males and 3 females) from the intervention group were recruited using a convenience sampling approach. Three broad categories formed the code template: views on their experiences of using Imperative Health (Web-based program), views on Imperative Health*’s* website components (see Table 1) that support behavior change, and suggested improvements that Imperative Health should implement. Presented subsequently are some of the quotations used to demonstrate typical views within each code category.

#### Experiences Using Imperative Health (Web-Based Program)

All interviewees stated that they were keen to use this Web-based program to help them lose weight and manage their chronic condition. They found the initial setup of their Imperative Health accounts relatively straightforward:

It was very straightforward. I don’t think I had any difficulty at all with it.

Some of the interviewees perceived using the Web-based program as time consuming and quite burdensome; specifically, the tasks that involved uploading and manually entering measurements as well as working through the weekly feedback:

I don’t know whether people who would be employed full time would have enough time to do that...If you’re running out to work in the morning and you have to be out for 7 o’clock, you’re not going to be standing there weighing yourself, and again, when you come back home again, typing in what you have done.

If you wanted a quick consult, it was taking you 10 minutes to get through...

#### Website Components That Support Behavior Change

The majority of interviewees found the personalized targets (weight loss, physical activity, and dietary targets) provided by the Web-based program realistic and motivating:

It did give me the motivation to say, “Right, I’m supposed to go walking 60 minutes a day. I’ll try and keep to that target of 60 minutes a day.” They weren’t tough; the calories I was being allowed were okay...

The majority of interviewees were not impressed with the Web-based program planning components (meal planner and exercise schedule). Accessibility issues and aversions to the recommended foods in the meal plans were commented upon:

If it had of been prepackaged food or something that I would have actually liked but there was none of the stuff that really appealed to me. So I never used the meal planner...

But they were all foods that were for supermarkets, say, in England and a lot of the stuff that you wouldn’t get here maybe.

All the interviewees felt that the Web-based program’s self-monitoring components (weighing scales and accelerometer activity band—data from these devices were uploaded onto colored charts to track progress) helped them to evaluate their progress and at the same time acted as facilitators for motivating them to keep continuing toward their targets.

With regard to the weight one, it encouraged you to do better, because it showed if you were flattening out or, at worst, going the wrong way off your target.

I found the activity useful because when I would sync up at the end of the week I would have a look and say “I was low on Tuesday and Wednesday this week. I’ll maybe do a boost on Friday. I’ll go for an hour and a half walk just to make sure my average for the week is up.” So I found that a little bit, slightly motivating.

All interviewees provided negative feedback regarding the dietary self-monitoring element of this Web-based program (food diary). They felt the process was time consuming and burdensome as a result of having to look up all the calories of the foods they consumed and then enter them manually into the food diary:

I found getting the nutritional values of things awkward because you had to go into a separate wee thing in the background and then you had to write it down and then you go back to something else.

Most of the interviewees claimed they did not use the component for monitoring their clinical measurements, as they were unable to get these health risk factors measured regularly:

...The average person doesn’t have that information. I might get that done twice a year.

The majority of the participants were not impressed with the automated feedback and coaching sessions provided weekly. They felt that it was too generic and repetitive; hence, not encouraging or constructive:

The other thing that irritated me intensely is the standard messages that you would get at every stage of the bloody feedback! I suppose it’s a computer system, what can you expect, but I just got cheesed off because it said the same thing all the time.

It was more generic in the sense. They were just basically saying “we haven’t got enough information” or “you have not met your target.”

Most participants stated that they did browse the community forum but did not contribute anything. They generally felt that there was not enough activity:

I occasionally dipped in and out to see what it was but there was very little action or interest, and I don’t get involved in anything like that at all.

#### Suggested Improvements

A common suggestion for improvement was more personalized interaction and feedback specifically from a human rather than an automated machine because this may provide them with more focus and motivation. This would be in-line with the full Imperative Health service that has physiologists supporting participants by telephone and email.

...some more personalized interaction in terms of somebody perhaps phoning you on your mobile to give you a kick-start or perhaps an email...

I tried at the start, and because there is not actually a person involved in it you’re not worried about what the machine tells you then, you don’t care what it says to you. So you go off track a wee bit...

## Discussion

### Weight Loss (Primary and Secondary Outcome)

In comparison to a “true” control group, access to a Web-based program resulted in significantly greater weight loss in the intervention group after 3 and 6 months. However, longer-term follow-up indicated that the difference in weight loss between the intervention and control group was not sustained at 12 months. The reasons for this were twofold: weight regain in the intervention group between the 6- and 12-month time point and an increase in weight loss in the control group over the same time period. In terms of clinically significant weight loss (weight loss of ≥5% of baseline body weight), significantly more participants in the intervention group compared to the control group lost 5% or more of their baseline body weight at 3 months (34% vs 3%, *P*<.001) and at 6 months (41% vs 18%, *P*=.047), but not at 12 months (22% vs 21%, *P*=.95).

### Engagement, Nonusage Attrition, and Attrition

This study was designed to evaluate this Web-based program in a real-life setting to observe real levels of engagement and their relationship with weight loss; hence, no instructions were given to participants regarding how often they should log in to use the website and the self-monitoring devices. The Imperative Health program does encourage daily engagement by allowing the upload of daily weight and physical activity data, captured by the accelerometer activity band and by the entry of daily food diaries. Some studies have suggested that an unstructured self-care approach may limit the potential benefit of Internet programs [30,31]; however, prescriptive dosage studies are likely to represent efficacy rather than effectiveness and do not help to understand the likely true public health impact of these novel modes of delivery. Studies that have provided dosage instructions have found positive effects. For example, participants that comply with the dosage instructions tend to lose significantly more weight than noncompliers [14,32-34]. The majority of these prescriptive dose studies, however, were conducted over the short term (6 months or less). Sustaining engagement levels in the long term is undoubtedly more of a challenge. Weight change (3 months) in this study had a positive moderate correlation with the number of log-ins and weight uploads, but engagement levels tended to diminish with time, particularly after 6 months. Web-based programs in general tend to have problems with long-term sustainability and nonusage attrition tends to be a common characteristic that increases steadily over time [2,31]. Participants are likely to disengage over time, perhaps due to motivational issues and, particularly, if they are failing to lose weight or have reached a plateau [35]. Furthermore, depending on the Web-based program itself and what it has to offer in terms of interactivity and level of intensity, participants may simply get bored and lose interest in the Web-based program.

Attrition rates are generally high in Web-based weight loss studies and have been reported to range between 0% and 70%, with a mean attrition rate of 22.5% [7]. Furthermore, attrition rates have been reported to be higher within the Web-based intervention group [30,32,36-38] relative to the control group, as was the case in this study.

Interactivity is essential for high engagement and low attrition; furthermore, it is well-accepted that Web-based programs with enhanced interactive features promote greater weight reduction than those that provide information only [5]. The Web-based program described in this study encompassed an interactive design by encouraging self-monitoring and providing automated feedback, yet high levels of attrition and long-term disengagement levels were evident. Incorporation of more individualized personal support rather than automated feedback may have helped engagement levels, particularly at the 6-month juncture. The majority of participants who took part in an interview suggested that the addition of more personalized interaction, by a phone call or email, rather than an automated machine providing standard feedback would be more motivating and help preserve their interest to keep using the program. Similar studies evaluating the effect of Web-based programs on weight loss have reported higher effect sizes and usage when face-to-face contact is incorporated into the intervention [4]. This would be in-line with the complete Imperative Health service; however, it was important to assess the Web-based program on its own to understand its specific contribution.

Incorporation of Web-based programs into traditional care pathways for weight loss has generally taken the approach of comparing standard health care to standard health care plus a Web-based program over a defined period of time. An alternative model of care that may be worth further investigation is to use Web-based programs for initiation of weight loss and then add in further interventions rather than using Web-based programs alongside other interventions from the outset. Addition of more interpersonal interventions at the later stage would perhaps encourage sustained behavior change, prevent attrition from the 6-month time point onward, and support the weight loss maintenance stage. Such a model has particular relevance for health care systems. For example, waiting lists to be seen by dieticians in the UK National Health Service can be many weeks; referral to use a Web-based program during this time would be a useful way of initiating weight loss and may be particularly appealing for patients who do not feel comfortable attending weight loss groups.

### Secondary Outcomes

In terms of cardiovascular risk, between-group analyses demonstrated that the intervention group significantly improved their BMI and waist circumference at 3 months and 6 months and their total cholesterol and triglycerides from baseline to 3 months in comparison with the control group; however, these significant between-group changes were not sustained at 12 months. The Web-based program did provide a self-monitoring tool for tracking blood pressure, lipids, and lipoprotein levels, but intervention group participants did not avail of this part of the program. When this was discussed at the interview sessions, the majority of participants stated that the main reason they did not access this part of the program was because they were not able to have these risk factors measured regularly. This is a general disadvantage of Web-based programs that do encourage the monitoring of other health risk factors, but do not provide the means to conduct the measurements at the participant’s own convenience.

It was evident that the intervention group adopted healthier behaviors specifically in the short term. The significant increase in time spent exercising moderately and above (>3.5 METs) and the decrease in energy intake observed in the intervention group in comparison to the control group was likely to be attributable to the self-monitoring components of the Web-based program. Usages of these self-monitoring features were also correlated with short-term weight change (baseline to 3 and 6 months). Physical activity levels were not sustained in the longer term and the number of weekly physical activity uploads notably decreased between 6 and 12 months. These findings are consistent with those already reported in the literature [39,40]. A systematic review examining the effects of self-monitoring diet, physical activity, and weight on weight loss [39] found a consistent and positive significant association between the frequency of the self-monitoring behaviors and weight loss compared to less frequent self-monitoring. It was also reported in this review that a gradual decline over time in adherence to self-monitoring weight management behaviors is common [39].

### Strengths and Limitations

The strengths of this study included its robust study design, the objectively measured primary outcome, and the mixed-method research approaches (qualitative and quantitative) used throughout the evaluation process. Furthermore, this study included a true control group. The majority of studies in this area tend to use a minimal support group to boost recruitment and decrease attrition; however, this may attenuate the relationship between groups. This study was conducted within a real-life setting and participants were not provided with strict instructions as to how often they should use the program; therefore, making the results more generalizable to overweight populations accessing these Web-based programs at home for their own self-care.

This study did have some limitations, for example, all participants had contact with the researcher during clinical assessments and knew this was a weight loss study, which in itself may have triggered a behavior change response and the “Hawthorne effect” [41] appears to be evident within the control group. The researcher, however, did not give any advice during the assessment period to either group.

Issues of attrition or loss to follow-up and nonusage attrition steadily increased over time, but this phenomenon is commonly reported in the literature in relation to weight loss management [35] and is not unique to Web-based programs. From a scientific perspective, attrition and nonusage attrition can impact on the likelihood of detecting a difference between groups when evaluating the treatments over longer periods of time; from a clinical perspective, it highlights the challenge of maintaining interest, motivation, and weight loss in the medium to long term. The increased attrition over the 12-month intervention period diminished the power of the study to detect a difference in change in weight loss and other endpoints between the intervention and control group in the longer term. However, the difference in weight change between the 2 groups at 12 months was very small and did not provide support for the Web-based program having a clinically significant advantage for long-term weight loss relative to a true control (usual care).

Owing to the routes of recruitment and the fact that people volunteered themselves for this study, the majority of the sample was from a higher social economic background. This could have potentially affected levels of engagement and attrition. This is not unique to this study, but does suggest the sample is not likely to be entirely representative of the general overweight and obese population. This will need to be borne in mind when considering the potential wider- or larger-scale impact of the Web-based behavior change intervention.

### Conclusions

This study provides evidence that this Web-based program can be used to initiate clinically relevant weight loss of 5% or more and promote improvements in total cholesterol and triglyceride concentrations in the short term (3-6 months) in comparison with usual care. However, these changes were not sustained in the longer term (up to 12 months) and this appeared to correspond with a general decline in usage of the Web-based program over time. The fact that the study was powered on weight loss at the 3-month juncture and the high attrition rates at the 12-month time point in the intervention group, could have also prevented significant differences between the groups being identified, specifically at the later time point. Nevertheless, results of this study highlight a need to augment Web-based programs with further interventions after 6 months of usage, such as phone, email, or face-to-face support, to enhance engagement, prevent relapses, and encourage maintenance of weight loss in the longer term. The effectiveness and cost-effectiveness of such a model of weight management is worth further exploration

## Appendix

Multimedia Appendix 1 CONSORT-EHEALTH checklist V1.6.2 [10].

## Abbreviations

BMI: body mass index
BOCF: baseline observation carried forward
CRP: C-reactive protein
CVD: cardiovascular disease
GPPAQ: General Practice Physical Activity Questionnaire
HDL: high-density lipoprotein
ITT: intention-to-treat
MET: Metabolic Equivalent Task
NE-SEC: National Statistics Socio-Economic Classification
QUB: Queen’s University Belfast
RCT: randomized controlled trial
RPAQ: Recent Physical Activity Questionnaire
