# Supplementary material for: Effect of a Web-Based Behavior Change Program on Weight Loss and Cardiovascular Risk Factors in Overweight and Obese Adults at High Risk of Developing Cardiovascular Disease: Randomized Controlled Trial
Source: J Med Internet Res. 2015 Jul 16;17(7):e177. doi: 10.2196/jmir.3828 (PMC4527000; doi:10.2196/jmir.3828)
Supplement: Multimedia Appendix 1 [file jmir_v17i7e177_app1.pdf]

CONSORT-EHEALTH Checklist V1.6.2 Report

(based on CONSORT-EHEALTH V1.6), available at [http://tinyurl.com/consort-ehealth-v1-6].

3828

Date completed

8/25/2014 10:06:08

by

Sinead Watson

Effect of a unique web-based behaviour change program on weight loss and cardiovascular risk factors in overweight and obese adults at high risk of developing cardiovascular disease. A randomised controlled trial

TITLE

1a-i) Identify the mode of delivery in the title

"Effect of a unique web-based behaviour change program on weight loss and cardiovascular risk factors in overweight and obese adults at high risk of developing cardiovascular disease. A randomised controlled trial."

1a-ii) Non-web-based components or important co-interventions in title

No, this paper does not mention non-web-based components in the title as these were not part of the intervention.

1a-iii) Primary condition or target group in the title

"Effect of a unique web-based behaviour change program on weight loss and cardiovascular risk factors in overweight and obese adults at high risk of developing cardiovascular disease. A randomised controlled trial".

ABSTRACT

1b-i) Key features/functionalities/components of the intervention and comparator in the METHODS section of the ABSTRACT

The abstract mentions some of the key features and components of the intervention. The comparator group were not provided with an intervention; instead, they were asked to continue with their usual self and medical care.

"Group one were provided with the web-based component of a service called Imperative Health. This WBP supports positive dietary and physical activity changes and assists in managing weight and other cardiovascular risk factors.
It combines objective monitoring of weight and physical activity with automated, tailored feedback. Group two were asked to continue with their usual self-care."

1b-ii) Level of human involvement in the METHODS section of the ABSTRACT

Human involvement was not part of the intervention.

"Imperative Health is a service that consists of a web-based program and human (email and telephone) support that assists in lifestyle change; however, for this particular study only the web-based program was evaluated in order to determine its specific impact, i.e. the human support (telephone and email) component of the service was removed."

1b-iii) Open vs. closed, web-based (self-assessment) vs. face-to-face assessments in the METHODS section of the ABSTRACT

The abstract does not mention how the participants were recruited but does mention that assessments were carried out face-to-face.

1b-iv) RESULTS section in abstract must contain use data

"Sixty-five overweight/obese adults at high risk of CVD were randomly allocated to one of two groups. Group one (n=32) were provided with the web-based component of a service called Imperative Health. Group two were asked to continue with their usual self-care (n=33)."

"Retention rates for the intervention and control groups at 3 months were (78% vs 97%), at 6 months (66% vs 94%) and at 12 months (53% vs 88%), respectively."

1b-v) CONCLUSIONS/DISCUSSION in abstract for negative trials

"This study indicates that this WBP can be used to initiate and achieve clinically relevant weight loss and lower CVD risk but highlights a need for augmenting WBPs with further interventions, such as telephone, email or in-person support in the longer term to maintain these changes."

INTRODUCTION

2a-i) Problem and the type of system/solution

"The prevalence of obesity has been increasing progressively throughout the world [1]. Identifying effective and cost-effective treatment and prevention strategies is a top priority for all healthcare systems. Over the past few decades the internet has increasingly been used to deliver behavioural modification programs owing to its easy accessibility and anonymity, potential for wide reach and penetration, and its ability to provide a source of continuous support to large segments of the population [2,3,4]."

"Therefore, the aim of this study was to evaluate the effects of an interactive web-based component of a service called Imperative Health on weight loss (primary outcome) and CVD risk factors (secondary outcomes) in an overweight and obese population at high risk of CVD using a randomised controlled design and a 'true control' group. It was hypothesised that weight loss would be greater in the WBP intervention group compared to the usual care control group."

2a-ii) Scientific background, rationale: What is known about the (type of) system

"There is growing evidence suggesting that the internet may be a viable medium for encouraging weight loss. However, several systematic reviews and meta-analysis, conducted in this area have found it difficult to draw definitive conclusions regarding its effectiveness owing to heterogeneity in study designs, methods employed and the lack of 'true control' groups used [5,6,7,8,9]. Most of the evidence to date comes from randomised controlled trials (RCTs) conducted in the USA. Many only included short-term follow-up and lacked 'true control' groups (no support provided), making it challenging to accurately evaluate the true effectiveness of web-based programs (WBPs). Instead, minimal support groups are often employed to help boost recruitment and decrease attrition, although this approach may attenuate the relationship between groups and limits the ability of the findings to inform cost-effectiveness and healthcare models. Research is also limited regarding the effect of these WBPs on other health outcomes that co-exist with weight loss, such as cardiovascular disease (CVD) risk factors."

METHODS

3a) CONSORT: Description of trial design (such as parallel, factorial) including allocation ratio

"The aim of this study was to evaluate the effects of an interactive web-based component of a service called Imperative Health on weight loss (primary outcome) and CVD risk factors (secondary outcomes) in an overweight and obese population at high risk of CVD using a randomised controlled design and a 'true control' group. It was hypothesised that weight loss would be greater in the WBP intervention group compared to the usual care control group."

3b) CONSORT: Important changes to methods after trial commencement (such as eligibility criteria), with reasons

No changes.

3b-i) Bug fixes, Downtimes, Content Changes

There were no changes to the ehealth system or unexpected events.

4a) CONSORT: Eligibility criteria for participants

"Participants were eligible if they were aged over 18 years, had a BMI between 27 to 40 kg/m2, were inactive or moderately inactive assessed by the General Practice Physical Activity Questionnaire (GPPAQ) [11] and had one or more CVD risk factor: high blood pressure ≥ 140/90 mmHg, cholesterol ≥ 5.0 mmol/l or type 2 diabetes. All participants were required to have access to the internet, email, and a telephone and were asked not to participate in another behavioural change weight loss program throughout the study period. Participants were excluded if they had established CVD, type 1 diabetes, were pregnant or consumed excessive amounts of alcohol. Computer literacy was not assessed."

4a-i) Computer / Internet literacy

Computer literacy was not part of the eligibility criteria and was not assessed. However, participants were required to have access to the internet and email to take part in the study.

4a-ii) Open vs. closed, web-based vs. face-to-face assessments:

"Participants were recruited from April to December 2011 using posters in public places in the greater Belfast area, and intranet advertisements via staff updates in the Belfast Health and Social Care Trust and Queen's University Belfast (QUB). Patients from the Regional Centre for Diabetes and Endocrinology at the Royal Victoria Hospital Belfast were also sent a letter informing them about the study."

"Assessments were carried out face-to-face at the Regional Centre for Endocrinology and Diabetes at the Royal Victoria Hospital Belfast at baseline, 3 months, 6 months and 12 months."

4a-iii) Information giving during recruitment

"All participants provided written informed consent." Procedures of obtaining informed consent were not mentioned in the manuscript.

Written informed consent was obtained as follows:
Potential participants that were interested in the study were provided with a consent form and a study information sheet which explained the overall objectives of the study and what it would entail; assessments, time commitment, any risks involved as well as accentuating anonymity and confidentiality of results. After a cooling off period of 72 hours from learning about the study and obtaining the information sheet, participants were contacted by the researcher to determine whether or not they would like to take part in the study. All participants screened for eligibility provided written informed consent which was obtained by the researcher face-to-face. All participants were informed that it was a voluntary study and that they could freely withdraw from the study at any time and that their medical care or legal rights would not be affected.

4b) CONSORT: Settings and locations where the data were collected

"Assessments were carried out face-to-face at the Regional Centre for Endocrinology and Diabetes at the Royal Victoria Hospital Belfast at baseline, 3 months, 6 months and 12 months."

4b-i) Report if outcomes were (self-)assessed through online questionnaires

Outcomes for this study were not (self-) assessed through online questionnaires; they were assessed face-to-face.

"Assessments were carried out face-to-face at the Regional Centre for Endocrinology and Diabetes at the Royal Victoria Hospital Belfast at baseline, 3 months, 6 months and 12 months."

4b-ii) Report how institutional affiliations are displayed

Institutional affiliations were not displayed on the web-based program, they were displayed on the participant information sheet and consent form.

5) CONSORT: Describe the interventions for each group with sufficient details to allow replication, including how and when they were actually administered

5-i) Mention names, credential, affiliations of the developers, sponsors, and owners

The owner of the web-based program (Imperative Health) is specified as AXA PPP Healthcare in the manuscript.

5-ii) Describe the history/development process

"Previous versions of this WBP have been evaluated by Hurling et al. [16,17] and Ware et al. [18]; this program has since been modified to be more relevant to individuals that have independent risk factors for CVD such as hypertension, dyslipidaema (high cholesterol and triglycerides) and type 2 diabetes."

5-iii) Revisions and updating

There were no major changes to the intervention during the evaluation process.

5-iv) Quality assurance methods

Not included in the manuscript.

5-v) Ensure replicability by publishing the source code, and/or providing screenshots/screen-capture video, and/or providing flowcharts of the algorithms used

Screenshots of the web-based program were taken by the researcher and are available in Multimedia Appendices 2, 3 & 4.

5-vi) Digital preservation

The URL of the application is provided in the references section of the manuscript and is archived at web citation.org. Screenshots of the web-based program were taken by the researcher and are available in Multimedia Appendices 2, 3 & 4 in the manuscript.

5-vii) Access

"At the end of the baseline appointment the intervention group participants were provided with the Imperative Health package that contained the self-monitoring devices (Bluetooth enabled weighing scales and an accelerometer activity band) and basic written instructions to set up an online account at home. To access the online program participants were instructed to go to the Imperative health website [19] and enter a unique code to create their own personal password-protected free account. To complete registration and to enable the set up of the monitoring devices participants were advised to follow the online instructions."

5-viii) Mode of delivery, features/functionalities/components of the intervention and comparator, and the theoretical framework

"Imperative Health is a service, owned by AXA PPP Healthcare Limited, that consists of a WBP and human (email and telephone) support that assists in lifestyle change; with a particular focus on improving diet and nutrition, increasing physical activity and managing weight and other CVD risk factors. It combines objective monitoring of weight and physical activity with automated, tailored feedback and support by physiologists by telephone and email."

"The WBP encompasses supportive components to help facilitate lifestyle change (See Table 1). These WBP components were developed based on well recognised behaviour change strategies such as: planning, self-monitoring, goal setting and structured feedback, which were all used within the Diabetes Prevention Program [20] to promote weight loss."

5-ix) Describe use parameters

"This current study wanted to evaluate this WBP in a real life setting in order to determine realistic levels of engagement and their relationship with weight loss, therefore, no instructions were provided by the researcher as to how often the participants should login to use the website components and the self-monitoring devices. The WBP, however, does itself encourage daily engagement by allowing the upload of daily weight and physical activity data and by the entry of daily food diaries."

5-x) Clarify the level of human involvement

This was an evaluation of a web-based behaviour change program, human support was not part of the intervention. Enrolment as well as all the assessments, however, were carried out face-to-face.

"For this particular study only the WBP component of the service was evaluated in order to determine its specific impact, i.e. the human support (telephone and email) component of the service was removed for the purposes of this trial."

"Assessments were carried out face-to-face at the Regional Centre for Endocrinology and Diabetes at the Royal Victoria Hospital Belfast at baseline, 3 months, 6 months and 12 months."

5-xi) Report any prompts/reminders used

"Text messages or emails were sent daily and weekly to help remind participants to login and to weigh themselves."

5-xii) Describe any co-interventions (incl. training/support)

No other interventions (training/support) were provided in addition to the targeted ehealth intervention.

"At the end of the baseline appointment the intervention group participants were provided with the Imperative Health package that contained the self-monitoring devices (Bluetooth enabled weighing scales and an accelerometer activity band) and basic written instructions to set up an online account at home. To access the online program participants were instructed to go to the Imperative health website [19] and enter a unique code to create their own personal password-protected free account. To complete registration and to enable the set up of the monitoring devices participants were advised to follow the online instructions. The intervention group was informed at the baseline appointment that if any problems occurred throughout the study period after the initial set-up regarding the technology they were instructed to contact Imperative Health rather than the researcher."

6a) CONSORT: Completely defined pre-specified primary and secondary outcome measures, including how and when they were assessed

"Assessments were carried out face-to-face at the Regional Centre for Endocrinology and Diabetes at the Royal Victoria Hospital Belfast at baseline, 3 months, 6 months and 12 months."

"The primary outcome for this study was between-group change in body weight (kg) at 3 months. Weight was measured, without shoes and in light clothing, to the nearest 0.1 kg using calibrated Salter 994 digital weighing scales (Salter Housewares Ltd, Tonbridge, UK)."

"Secondary outcomes were between-group change in weight loss at 6 and 12 months and between-group change in the following risk markers at each follow-up: Body mass index (BMI) calculated as weight (kg) divided by height (m) squared; height was measured to the nearest 0.1 cm using a Leicester portable height measure (CMS Weighing equipment Ltd, London, UK); waist circumference was measured to the nearest 0.5 cm using a tape measure at the middle point between the lower rib margin and iliac crest at normal expiration. Blood pressure (mmHg) was measured using an automated Omron M3 sphygmomanometer (Omron Healthcare, Hoofddorp, The Netherlands). Fasting serum lipid profile included measurements of total cholesterol, high density lipoprotein (HDL) cholesterol and triglycerides and were measured using standard assays on an automated ILab 600 Chemistry system (Instrumentation Laboratory, Cheshire, UK). Plasma hs-CRP (CRP) was measured using an ultra-sensitive assay (quantex CRP ultra-sensitive; Instrumentation Laboratory, Cheshire, UK) on an automated machine, (ILab 600 Chemistry System).
Dietary intake was assessed using a diet history interview [21], which is a retrospective dietary assessment method used to gather information regarding the habitual food intake of all the participants over the previous 3 months. The diet history method has been shown to have good repeatability in previous studies and is also able to pick up dietary changes over time [22]. Quantities of food and food portion sizes (household measures) were converted into weights (grams) by using Crawley's Food Portion Sizes (Food Standards Agency) [23]. The food type, preparation method if relevant, and weight of food were entered into a computerised food analysis database (WISP, Weighed Intake Software Program; Tinuviel Software, Warrington, UK). For the purpose of this current study total daily energy intake (kcal) was calculated. Physical activity was assessed using the validated Recent Physical Activity Questionnaire (RPAQ) [24]. Participants were asked to provide descriptions of their habitual physical activity performed in four domains: home, work, travel and recreation over the last four weeks. For the purpose of this current study time (min/day) spent participating in moderate and vigorous activities (> 3.5 METs) was calculated."

6a-i) Online questionnaires: describe if they were validated for online use and apply CHERRIES items to describe how the questionnaires were designed/deployed

Online questionnaires were not used. All assessments were carried out face-to-face.

6a-ii) Describe whether and how "use" (including intensity of use/dosage) was defined/measured/monitored

"Data on frequency of logins, the total number of completed food diaries and the number of weight and physical activity uploads from the monitoring devices were provided by Imperative Health and were used to determine level of engagement."

6a-iii) Describe whether, how, and when qualitative feedback from participants was obtained

Qualitative feedback was obtained from the participants.

"To gain in-depth feedback on the intervention group's experiences of using the WBP these participants were asked if they would be willing to take part in an interview conducted by the researcher towards the end of the study. This was an optional part of the study and for this reason a convenience sampling technique was utilised. The interviews were conducted between July and August 2012, in the Centre for Public Health, QUB, within an informal setting and lasted ~25–30 minutes.
Semi-structured open-ended questions were used throughout to ensure that a consistent approach was utilised. The researcher used a style of probing to extract more information or clarify meaning."

6b) CONSORT: Any changes to trial outcomes after the trial commenced, with reasons

No changes to trial outcomes occurred after trial commencement.

7a) CONSORT: How sample size was determined

7a-i) Describe whether and how expected attrition was taken into account when calculating the sample size

"Based on a standard deviation of weight loss at 3 months of 3.0 kg, observed in a number of internet-based weight loss studies in the literature [12,13,14,15], it was estimated that a sample size of 60 (30 per group) would give the study 90% power at the 5% significance level to detect a difference of 2.6 kg between groups at the 3 month follow-up. Allowing for a 10% drop out rate at the first 3 month follow-up, it was aimed to recruit 66 participants."

7b) CONSORT: When applicable, explanation of any interim analyses and stopping guidelines

No interim analyses or stopping guidelines.

8a) CONSORT: Method used to generate the random allocation sequence

"Participants were randomly allocated to one of two parallel groups (1:1 allocation ratio) using a block randomisation approach (block size = 10) with computer-generated numbers (figure 1). A researcher independent from the study prepared the randomisation schedule. Opaque sealed envelopes were used to conceal the sequence until groups were allocated. Participants were enrolled by the researcher (SW), who was unaware of the randomisation schedule until after the baseline assessments when the sealed envelope containing the allocation outcome was opened by the participant."

8b) CONSORT: Type of randomisation; details of any restriction (such as blocking and block size)

"Participants were randomly allocated to one of two parallel groups (1:1 allocation ratio) using a block randomisation approach (block size = 10) with computer-generated numbers (figure 1)."

9) CONSORT: Mechanism used to implement the random allocation sequence (such as sequentially numbered containers), describing any steps taken to conceal the sequence until interventions were assigned

"Participants were randomly allocated to one of two parallel groups (1:1 allocation ratio) using a block randomisation approach (block size = 10) with computer-generated numbers (figure 1). A researcher independent from the study prepared the randomisation schedule. Opaque sealed envelopes were used to conceal the sequence until groups were allocated. Participants were enrolled by the researcher (SW), who was unaware of the randomisation schedule until after the baseline assessments when the sealed envelope containing the allocation outcome was opened by the participant."

10) CONSORT: Who generated the random allocation sequence, who enrolled participants, and who assigned participants to interventions

A researcher independent from the study generated the random allocation sequence and prepared the opaque sealed envelopes.

"Participants were recruited and enrolled by the researcher, who was unaware of the randomisation schedule until after the baseline assessments when the sealed envelope containing the allocation outcome was opened by the participant."

11a) CONSORT: Blinding - If done, who was blinded after assignment to interventions (for example, participants, care providers, those assessing outcomes) and how

11a-i) Specify who was blinded, and who wasn't

Giving the nature of the intervention it was not possible to blind the participant or the researcher.

"With only one researcher on the ground, it was not possible to blind the researcher or participants to group allocation but laboratory analysis was performed blind.'

11a-ii) Discuss e.g., whether participants knew which intervention was the "intervention of interest" and which one was the "comparator"

Not mentioned in the manuscript.

Participants were informed that they could be allocated to the web-based behaviour change group or to the usual care control group when they were provided with a participant information sheet telling them about the study and again when obtaining informed consent.

11b) CONSORT: If relevant, description of the similarity of interventions

Not relevant for this trial.

12a) CONSORT: Statistical methods used to compare groups for primary and secondary outcomes

"All analyses were performed using SPSS for Windows version 21.0 (SPSS Inc, Chicago, IL). Results are expressed as mean and standard deviation for normally distributed variables and median and inter-quartile range for variables that did not satisfy normality criteria. Categorical data are expressed as frequencies and percentages. To compare baseline characteristics between the control and intervention groups, for continuous variables, the appropriate parametric (Independent samples t-test) and non-parametric tests (Mann Whitney U test) were utilised. For categorical variables the Chi-square test was used. Between-group differences in the primary outcome (weight change at 3 months) and secondary outcomes from baseline to 3 months, 6 months and 12 months were investigated using the analysis of covariance (ANCOVA) adjusted for baseline measurements [26]. Analyses were carried out by an intention-to-treat (ITT) approach using a single imputation method (baseline observation carried forward, BOCF) to deal with missing data and losses to follow-up [27], and a complete-case analysis on weight change was conducted using information on all individuals with available data at each time point. C-reactive protein, triglycerides and physical activity distributions were skewed and therefore log transformed. Adjusted differences in log-transformed means between groups from ANCOVA were converted to, and reported as, ratios of geometric means and 95% confidence intervals. Within-group changes (intervention or control) in weight loss were analysed using paired sample t-test. As the WBP usage data was not normally distributed, Spearman correlations were performed to investigate the relationship between weight change and WBP usage at each time point (intervention group only)."

12a-i) Imputation techniques to deal with attrition / missing values

"Analyses were carried out by an intention-to-treat (ITT) approach using a single imputation method (baseline observation carried forward, BOCF) to deal with missing data and losses to follow-up [27]."

12b) CONSORT: Methods for additional analyses, such as subgroup analyses and adjusted analyses

"Spearman correlations were performed to investigate the relationship between weight change and WBP usage at each time point (intervention group only)".

"Between-group differences in the primary outcome (weight change at 3 months) and secondary outcomes from baseline to 3 months, 6 months and 12 months were investigated using the analysis of covariance (ANCOVA) adjusted for baseline measurements [26]".

RESULTS

13a) CONSORT: For each group, the numbers of participants who were randomly assigned, received intended treatment, and were analysed for the primary outcome

A total of 65 participants were randomised to the intervention (n=32) or control (n=33) groups. All participants randomised received their allocated treatment. The primary outcome of this study was between-group change in body weight at 3 months. Thirty-two participants in the intervention group and 33 in the control group were analysed for this primary outcome.

Figure 1 in the manuscript shows the flow of participants throughout the trial; how many randomised, how many received intended treatment and how many were analysed for the primary outcome.

13b) CONSORT: For each group, losses and exclusions after randomisation, together with reasons

Figure 1 in this manuscript is a CONSORT flow diagram that shows the flow of participants through the trial (losses) and includes the reasons for losses.

13b-i) Attrition diagram

An attrition diagram was not provided. "Table 5 in the manuscript shows website utilisation (number of logins, food diary entries, weight uploads and physical activity uploads) from baseline to to 3 months, 6 months and 12 months (intervention group only)."

14a) CONSORT: Dates defining the periods of recruitment and follow-up

"Participants were recruited from April to December 2011 using posters in public places in the greater Belfast area, and intranet advertisements via staff updates in the Belfast Health and Social Care Trust and Queen's University Belfast (QUB). Patients from the Regional Centre for Diabetes and Endocrinology at the Royal Victoria Hospital Belfast were also sent a letter informing them about the study."

"Assessments were carried out face-to-face at the Regional Centre for Endocrinology and Diabetes at the Royal Victoria Hospital Belfast at baseline, 3 months, 6 months and 12 months."

14a-i) Indicate if critical "secular events" fell into the study period

No secular events.

14b) CONSORT: Why the trial ended or was stopped (early)

The trial was not stopped early.

15) CONSORT: A table showing baseline demographic and clinical characteristics for each group

Table 2 in the manuscript shows baseline demographic (gender and age) and clinical (anthropometric, blood pressure and lipid measurements) characteristics for the intervention and the control group.

15-i) Report demographics associated with digital divide issues

Table 2 in the manuscript reports demographics (age and gender) associated with the digital divide.

Computer/internet/ehealth literacy was not assessed; however, all participants were required to have access to the internet and email to take part in the study.

16a) CONSORT: For each group, number of participants (denominator) included in each analysis and whether the analysis was by original assigned groups

16-i) Report multiple "denominators" and provide definitions

Primary and secondary outcomes were analysed using an Intention-to-treat approach (Baseline observation carried forward). Refer to the tables in the manuscript.

16-ii) Primary analysis should be intent-to-treat

The primary outcome was analysed using an Intention-to-treat approach (Baseline observation carried forward). Refer to Table 2.

"Analyses were carried out by an intention-to-treat (ITT) approach using a single imputation method (baseline observation carried forward, BOCF) to deal with missing data and losses to follow-up [27]".

17a) CONSORT: For each primary and secondary outcome, results for each group, and the estimated effect size and its precision (such as 95% confidence interval)

Refer to Tables 2 and 3 for all results (primary and secondary outcomes for each group) with effect size and 95% CI.

17a-i) Presentation of process outcomes such as metrics of use and intensity of use

"Data on frequency of logins, the total number of completed food diaries and the number of weight and physical activity uploads from the monitoring devices were provided by Imperative Health and were used to determine level of engagement."

"Website utilisation data is presented in Table 5. Participants in the intervention group tended to login, upload their weight measurement and make food diary entries more frequently during the first 3 months of the intervention, website usage declined thereafter."

17b) CONSORT: For binary outcomes, presentation of both absolute and relative effect sizes is recommended

Binary outcomes are not assessed or reported in this manuscript.

18) CONSORT: Results of any other analyses performed, including subgroup analyses and adjusted analyses, distinguishing pre-specified from exploratory

Website usage and weight change (Intervention group only)
"Correlation analyses (Table 6) demonstrated that weight change from baseline to the 3 month follow-up was significantly positively related to the number of logins (P=.04) and the number of weight uploads (P=.007) at 3 months. A positive relationship was observed between weight change from baseline to 6 months and the amount of physical activity uploads over the same time period (P=.048). The number of daily food diaries entered was not related to weight change throughout the course of the study."

18-i) Subgroup analysis of comparing only users

Website usage and weight change (Intervention group only)
"Correlation analyses (Table 6) demonstrated that weight change from baseline to the 3 month follow-up was significantly positively related to the number of logins (P=.04) and the number of weight uploads (P=.007) at 3 months. A positive relationship was observed between weight change from baseline to 6 months and the amount of physical activity uploads over the same time period (P=.048). The number of daily food diaries entered was not related to weight change throughout the course of the study."

19) CONSORT: All important harms or unintended effects in each group

Harms or untended effects did not occur in either the intervention or control group.

19-i) Include privacy breaches, technical problems

There were no privacy breaches or technical problems.

19-ii) Include qualitative feedback from participants or observations from staff/researchers

"To gain in-depth feedback on the intervention group's experiences of using the WBP these participants were asked if they would be willing to take part in an interview conducted by the researcher towards the end of the study. This was an optional part of the study and for this reason a convenience sampling technique was utilised."

"A total of seven participants (four males and three females) from the intervention group were recruited using a convenience sampling approach. Three broad categories formed the code template: views on their experiences of using Imperative Health (WBP), views on Imperative Health's website components (see Table 1) that support behaviour change and suggested improvements that Imperative Health should implement. Below are some of the quotations used to demonstrate typical views within each code category."

DISCUSSION

20) CONSORT: Trial limitations, addressing sources of potential bias, imprecision, multiplicity of analyses

20-i) Typical limitations in ehealth trials

"This study did have some limitations, for example, all participants had contact with the researcher during clinical assessments, and knew this was a weight loss study, which in itself may have triggered a behaviour change response, and this 'Hawthorne effect' [40] appears to be evident within the control group. The researcher, however, did not give any advice during the assessment period to either group. Issues of attrition or loss to follow-up and non-usage attrition steadily increased over time, but this phenomenon is commonly reported in the literature in relation to weight loss management [34] and is not unique to WBPs. From a scientific perspective, attrition and non-usage attrition can impact on the likelihood of detecting a difference between groups when evaluating the treatments over longer periods of time, from a clinical perspective it highlights the challenge of maintaining interest, motivation and weight loss in the medium to long term."

21) CONSORT: Generalisability (external validity, applicability) of the trial findings

21-i) Generalizability to other populations

"This study was conducted within a real life setting and participants were not provided with strict instructions as to how often they should use the program, therefore making the results more generalisable to overweight populations accessing these WBPs at home for their own self-care."

21-ii) Discuss if there were elements in the RCT that would be different in a routine application setting

"Incorporation of WBPs into traditional care pathways for weight loss has generally taken the approach of comparing standard healthcare with standard healthcare plus WBP over a defined period of time. An alternative model of care that may be worth further investigation is to use WBPs for initiation of weight loss and then add in further interventions, rather than using WBPs alongside other interventions from the outset. Addition of more interpersonal interventions, at the later stage would perhaps encourage sustained behaviour change, prevent attrition from the 6 month time point onwards and support the weight loss maintenance stage. Such a model has particular relevance for healthcare systems, for example, waiting lists to be seen by Dieticians in the UK National Health Service can be many weeks; referral to use a WBP during this time would be a useful way of initiating weight loss and may be particularly appealing for patients who do not feel comfortable attending weight loss groups."

22) CONSORT: Interpretation consistent with results, balancing benefits and harms, and considering other relevant evidence

22-i) Restate study questions and summarize the answers suggested by the data, starting with primary outcomes and process outcomes (use)

Study aim:
"The aim of this study was to evaluate the effects of an interactive web-based component of a service called Imperative Health on weight loss (primary outcome) and CVD risk factors (secondary outcomes) in an overweight and obese population at high risk of CVD using a randomised controlled design and a 'true control' group."

Summary of results
"In comparison to a 'true control' group, access to a WBP resulted in significantly greater weight loss in the intervention group after 3 and 6 months. However, longer-term follow-up indicated that the difference in weight loss between the intervention and control group was not sustained at 12 months."

"In terms of cardiovascular risk, between-group analyses demonstrated that the intervention group significantly improved their BMI and waist circumference at 3 months and 6 months and their total cholesterol and triglycerides from baseline to 3 months in comparison with the control group, however, these significant between-group changes were not sustained at 12 months."

"It was evident that the intervention group adopted healthier behaviours specifically in the short term. The significant increase in time spent exercising moderately and above (> 3.5 METs) and the decrease in energy intake observed in the intervention group in comparison with the control group was likely to be attributable to the self-monitoring components of the web-based program. Usages of these self-monitoring features were also correlated with short-term weight change (baseline to 3 and 6 months). Physical activity levels were not sustained in the longer term and the number of weekly physical activity uploads notably decreased between 6 and 12 months."

22-ii) Highlight unanswered new questions, suggest future research

"Results of this study indicate that this web-based program can be used to initiate and achieve clinically relevant weight loss and highlights a need to augment WBPs with further interventions after 6 months of usage, for example phone or email or face-to-face support, in order to prevent relapses and encourage maintenance of weight loss in the longer-term. The effectiveness and cost-effectiveness of such a model of weight management is worth further exploration."

Other information

23) CONSORT: Registration number and name of trial registry

ClinicalTrials.gov identifier: NCT01472276;
http://clinicaltrials.gov/ct2/show/study/NCT01472276

24) CONSORT: Where the full trial protocol can be accessed, if available

The full trial protocol has not been published.

25) CONSORT: Sources of funding and other support (such as supply of drugs), role of funders

"This study was supported by a PhD studentship provided by the Northern Ireland Department for Employment and Learning (DEL). Imperative Health (AXA PPP Healthcare) provided the web-based program and monitoring devices without charge".

X26-i) Comment on ethics committee approval

"Ethical approval was obtained from the Office for Research Ethics Committees Northern Ireland."

"All participants provided written informed consent".

x26-ii) Outline informed consent procedures

The manuscript does state that written informed consent was obtained from all the participants face-to-face at the screening appointment. The manuscript doesn't outline the informed consent prodcedure. See below for the procedure.

Written informed consent was obtained as follows:
Potential participants who were interested in the study were provided with a consent form and a study information sheet which explained the overall objectives of the study and what it would entail; assessments, time commitment, any risks involved as well as accentuating anonymity and confidentiality of results. After a cooling off period of 72 hours from learning about the study and obtaining the information sheet, participants were contacted by the researcher to determine whether or not they would like to take part in the study. All participants screened for eligibility provided written informed consent which was obtained by the researcher face-to-face. All participants were informed that it was a voluntary study and that they could freely withdraw from the study at any time and that their medical care or legal rights would not be affected.

X26-iii) Safety and security procedures

"To access the online program participants were instructed to go to the Imperative health website [19] and enter a unique code to create their own personal password-protected free account". The intervention group was informed at the baseline appointment that if any problems occurred throughout the study period after the initial set-up regarding the technology they were instructed to contact Imperative Health rather than the researcher."

X27-i) State the relation of the study team towards the system being evaluated

"L Ware was involved in developing the nutrition and behaviour change elements of the web-based program. All other authors listed on the manuscript are distinct from the developers and sponsors of the intervention."

"This study was supported by a PhD studentship provided by the Northern Ireland Department for Employment and Learning (DEL). Imperative Health (AXA PPP Healthcare Ltd) provided the web-based program and monitoring
